# *Alternaria* Allergy and Asthma in Children

**DOI:** 10.3390/medicina61091639

**Published:** 2025-09-10

**Authors:** Angela Klain, Mattia Giovannini, Stefania Arasi, Simona Barni, Riccardo Castagnoli, Lucia Caminiti, Mariannita Gelsomino, Lucia Liotti, Carla Mastrorilli, Francesca Mori, Luca Pecoraro, Francesca Saretta, Michele Miraglia del Giudice, Elio Novembre

**Affiliations:** 1 Department of Woman, Child and General and Specialized Surgery, University of Campania Luigi Vanvitelli, 80138 Naples, Italy; angela.klain@studenti.unicampania.it (A.K.); michele.miragliadelgiudice@unicampania.it (M.M.d.G.); 2 Allergy Unit, Meyer Children’s Hospital IRCCS, 50139 Florence, Italy; simona.barni@meyer.it (S.B.); francesca.mori@meyer.it (F.M.); elio.novembre@unifi.it (E.N.); 3 Department of Health Sciences, University of Florence, 50139 Florence, Italy; 4 Translational Research in Pediatric Specialties Area, Division of Allergy, Bambino Gesù Children’s Hospital, IRCCS, 00165 Rome, Italy; stefania.arasi@opbg.net; 5 Department of Clinical, Surgical, Diagnostic and Pediatric Sciences, University of Pavia, 27100 Pavia, Italy; riccardo.castagnoli@unipv.it; 6 Pediatric Clinic, Fondazione IRCCS Policlinico San Matteo, 27100 Pavia, Italy; 7 Pediatric Unit, Department of Human Pathology in Adult and Development Age “Gaetano Barresi”, Allergy Unit, AOU Policlinico Gaetano Martino, 98124 Messina, Italy; lcaminiti@unime.it; 8 Pediatric Allergy Unit, Department of Life Sciences and Public Health, University Foundation Policlinico Gemelli IRCCS, Catholic University of the Sacred Heart, 00168 Rome, Italy; mariannita.gelsomino@guest.policlinicogemelli.it; 9 Pediatric Unit, Department of Mother and Child Health, Salesi Children’s Hospital, 60123 Ancona, Italy; lucialiotti@ospedaliriuniti.marche.it; 10 Pediatric Hospital Giovanni XXIII, Pediatric and Emergency Department, AOU Policlinic of Bari, 70126 Bari, Italy; carla.mastrorilli@policlinico.ba.it; 11 Pediatric Unit, Ospedale Vito Fazzi, ASL Lecce, 73100 Lecce, Italy; 12 Pediatric Department, Latisana-Palmanova Hospital, Azienda Sanitaria Universitaria Friuli Centrale, 33100 Udine, Italy; francesca.saretta@asufc.sanita.fvg.it

**Keywords:** *Alternaria*, *Alternaria alternata*, pediatric asthma, mold allergy, Alt a 1, allergen immunotherapy, component-resolved diagnostics

## Abstract

*Alternaria alternata* is one of the most clinically relevant fungal allergens in pediatric patients with respiratory allergies. Sensitization to this mold has increased in recent decades and is influenced by environmental exposure, geographic location, climate change, and genetic predisposition. In children, *Alternaria* spp. are strongly associated with the development and worsening of asthma and allergic rhinitis, often contributing to severe and difficult-to-control forms of the disease. The major allergen, Alt a 1, plays a central role in the immunopathogenesis of *Alternaria*-induced allergies and exhibits molecular features that allow cross-reactivity with other fungal species. Although *Alternaria* allergy is clinically relevant, its diagnosis remains challenging due to the variability and lack of standardization of fungal extracts. Therefore, it may be necessary to complement traditional diagnostic tools, such as skin prick testing and specific IgE measurement, with component-resolved diagnostics or, in selected cases, nasal provocation tests. Allergen immunotherapy (AIT) has shown promising results in the treatment of *Alternaria* allergy, particularly with the use of standardized Alt a 1-based extracts or chemically modified allergoids, which offer clinical benefits and immunological modulation. However, AIT is still underused in this context, partly because of the lack of widely available commercial products and long-term efficacy data in the pediatric population. This review provides a comprehensive overview of the current knowledge on the epidemiology, mechanisms, clinical implications, and treatment options related to *Alternaria* allergy in children, with the aim of supporting early recognition and tailored therapeutic strategies for this important, yet often underestimated, allergen.

## 1. Introduction

Fungi are a broad kingdom that includes various organisms such as yeasts, mushrooms, and molds. Molds are a specific multicellular type of fungi characterized by their fuzzy, filamentous appearance. They typically thrive on decaying organic matter and reproduce through spores, which can be spread through air or water. They can cause diseases via three main mechanisms: direct infection (mycoses), allergic reactions, and toxin production [1]. Fungi are increasingly being recognized as important contributors to the human exposome, the totality of environmental and biological exposures that influence health. They are present both externally, in outdoor and indoor environments, and internally, as part of the microbiome known as the microbiota, with considerable differences in their composition and health impact [2,3].

Molds are widespread in the environment; however, only a few hundred species act as opportunistic pathogens. Approximately 80 fungal genera induce type I IgE-mediated allergies, leading to conditions such as allergic rhinitis (AR) and asthma [4,5,6]. The most allergenic fungi belong to *Ascomycota* (e.g., *Alternaria*, *Cladosporium*, *Aspergillus*, *Penicillium*), followed by *Basidiomycota* (e.g., *Malassezia*) and *Zygomycota*. Other notable allergens include *Candida*, *Fusarium*, and *Rhizopus* [7].

*Alternaria* and *Cladosporium* are major outdoor allergens that disseminate in warm and dry air. Xerophilic species of *Aspergillus* and *Penicillium*, especially those found in indoor environments, contribute to allergic diseases by colonizing the respiratory tract [1]. Unlike pollen grains, *Alternaria* spores are much smaller, typically 3–8 µm in diameter, and can remain airborne for extended periods, allowing them to reach the lower airways upon inhalation [8]. Furthermore, the fungal spore season typically lasts twice as long as the pollen season, prolonging the risk of exposure for sensitized individuals. Despite their lower prevalence in ambient air than other fungal taxa, *Alternaria* spores have been shown to elicit the highest sensitization rates among atopic individuals and are strongly associated with asthma exacerbation, especially in children and during thunderstorm asthma events. Their small size, high allergenic potential, and prolonged presence in the air underline the importance of considering *Alternaria* spp. separately from pollen in allergy assessments and environmental control strategies [8].

Environmental prophylaxis is fundamental in the management of patients with mold allergies. Recommended measures include controlling the indoor moisture through proper building design and maintenance, repairing leaks, using dehumidifiers, and ensuring adequate ventilation. It is also advised to remove mold-contaminated materials, avoid wall-to-wall carpeting in damp areas, and maintain an indoor relative humidity of 30–50% [9,10].

## 2. Molds and *Alternaria* Sensitization

The prevalence of mold sensitization varies depending on environmental exposure and geographic location. A large-scale analysis conducted by a nationwide clinical laboratory in the United States assessed fungal sensitization in 1651,203 patients aged 0–85 y. Testing for 17 different fungal species indicated that 22% of patients had detectable specific IgE levels (>0.10 kUA/L) for at least one fungal allergen, whereas 13.7% showed sensitivity to more than two fungal allergens. The prevalence of sensitization varied by species, ranging from 7.4% to 18.6%, with the highest rates observed for *Candida albicans* (18.6%), *A. alternata* (16.6%), *Stemphylium herbarum* (14.9%), and *Aspergillus fumigatus* (14.2%). Overall, fungal sensitization is more common in urban areas and regions dominated by grasslands and prairies than in forested and woodland environments [11]. According to skin test results, the estimated prevalence of mold sensitization in Europe is approximately 5%, and *A. alternata* is one of the most common mold allergens in both adults and children [12,13,14]. Data on the general population are limited as most of the available evidence originates from allergy centers that monitor atopic individuals and/or patients with respiratory diseases. In a study by Mari et al. [15], 19.1% of 4962 patients aged 3–80 y with respiratory allergies were sensitized to at least one fungal species. *Alternaria* had the highest sensitization rate at 66.1%, followed by *Candida* (44.3%), *Trichophyton* (10.2%), *Aspergillus* (12.6%), *Cladosporium* (13.1%), *Penicillium* (8.1%), and *Saccharomyces* (7.4%) [15].

At the beginning of the 2000s, the European Community Respiratory Health Survey (ECRHS), which included 37 centers in 17 countries and approximately 11,355 adults, reported that sensitization to *Alternaria* among adults ranged from 0.2% to 14.4%, whereas *Cladosporium* sensitization varied between 0% and 11.9% [16,17].

In 2009, the Global Asthma and Allergy European Network (GA2LEN) conducted a multicenter, open-label study to assess clinically significant sensitization rates to various allergens across 14 European countries. The study included 3034 adults and children, with a median age of 33 y, suspected of having allergic conditions such as AR, asthma, atopic dermatitis, and food allergies. The prevalence of *Alternaria* sensitization varied across European countries. On a continental scale, the overall sensitization rate in Europe was 8.9% (95% CI: 7.9–9.9). However, substantial regional differences were reported. Greece had the highest prevalence of *Alternaria* sensitization (23.8%), followed by Germany (10.6%), Finland, and France (10.3%). Denmark reported a prevalence of 8.2%, whereas Austria and Hungary reported a prevalence of 6.5%. Belgium recorded 6.2%. At the lower end, the Netherlands, Portugal, and Switzerland had 5.5%, whereas Italy and Poland reported one of the lowest rates at 3.5%. The United Kingdom had the lowest prevalence at only 0.8% [18].

Of 2942 patients from 20 allergology centers in Italy, 10.4% showed sensitivity to *Alternaria*, although the rates varied widely between regions, from 1.8% in Turin to 29.3% in Cagliari. Of those sensitized, 79.7% had rhinitis and 53.3% had asthma. Only 12.1% of *Alternaria*-positive patients were monosensitized, whereas the majority also reacted to other allergens. Sensitization was more common in younger individuals, particularly those under 30 y, with a higher occurrence in males [19]. Similar results were observed in a 2021 study in Spain, in which data from 1156 patients recruited from 15 allergy departments across different bioclimatic regions were analyzed. The results showed that 20.2% of patients were sensitized to at least one of the five fungi tested, with *A. alternata* being the most common; sensitization was more prevalent in children (69.8%) and males (63%); most patients were also polysensitized to other allergens; regional differences were noted, with the highest prevalence observed in the Continental region and the lowest in the Oceanic region [20].

Sensitization to *A. alternata* has been reported in other parts of the world, with prevalence rates varying across populations. In Lima, Peru, 7% of the general adult population were sensitized to *A. alternata* [21]. In Africa, in an urban Gabonese population, 15% of 54 patients sensitized to inhalant allergens tested positive for *Alternaria* [22]. In Iran, a study of 1006 allergic patients found a sensitization rate of 5.3%, whereas in China, the prevalence rates were higher, with 14.9% of 397 asthmatic children showing sensitization, and an even greater rate of 44.9% among 1625 patients with AR [23,24]. Similarly, in Samara, Russia, 42.2% of 249 children diagnosed with AR and allergic asthma were sensitized to this mold [25] (Table 1).

### 2.1. Alternaria Characteristics and Allergens

The genus *Alternaria* comprises more than 300 fungal species. The most frequently and widely distributed *Alternaria* species is *A. alternata*, a ubiquitous filamentous fungus belonging to the *Pleosporaceae* family within the *Pleosporales* order. *Alternaria alternata* is a highly adaptable fungus with diverse roles as a saprophyte, plant pathogen, and human allergen [26]. It forms fast-growing black-pigmented colonies due to melanin synthesis, which enhances its resistance to environmental stressors and contributes to its survival and pathogenicity. *Alternaria alternata* produces various secondary metabolites, including mycotoxins, such as tenuazonic acid, alternariol, and alternariol monomethyl ether, which have been implicated in food contamination and pose potential health risks to humans and animals [26,27]. The fungus reproduces asexually via large, elongated, and transversely septate conidia that are produced in chains on the conidiophores [26]. These spores are efficiently dispersed under warm, dry, and windy conditions, reaching peak concentrations in late summer and early autumn, particularly in temperate regions [27].

Typically, airborne outdoor *Alternaria* spores can be detected from May to November, with an allergenic threshold of 100 spores/m^3^ [1,28]. Their concentrations are influenced by climate change, with rising temperatures and shifting weather patterns, potentially leading to higher airborne spore levels and extended seasons [28]. A 52-y study in England (1970–2021) indicated a strong link between rising seasonal temperatures and a 44% increase in *Alternaria* spore concentrations. A 1 °C pre-season rise caused a 2% earlier start. The authors suggest that by 2070, projected temperature increases (+1.3 °C to +5.1 °C) could further elevate *Alternaria* levels [29]. Similar findings were reported in a study conducted in Germany, where *Alternaria* sensitization rose substantially from 8.6% (1998–2007) to 11.7% (2008–2017), with a higher prevalence among younger individuals [30]. In contrast, in Korea, over 25 y (1998–2022) there has been a substantial decline in *Alternaria* spore concentrations due to increased rainfall over short periods, leading to longer dry seasons that limit fungal sporulation. Consequently, both spore concentrations and sensitization rates to *Alternaria* spp. decreased [31]. This tendency was also highlighted in Mexico, where over 11 y, *Alternaria* sensitization dropped from 21.4% in 2004 to 14.4% in 2015—a 7% reduction. In contrast, sensitization to other fungi, such as *Candida* and *Penicillium*, has increased [32]. These data suggest limited *Alternaria* adaptation to climate shifts, particularly compared to more adaptable pollen types.

Although *Alternaria* is primarily found outdoors, it can also colonize indoor environments with high humidity, such as damp walls, carpets, and poorly ventilated buildings, contributing to allergic sensitization and “sick building syndrome,” which refers to clinical manifestations (headaches, fatigue, and respiratory problems) experienced by building occupants, often due to poor indoor air quality [26,33]. The “Inner-City Asthma Study” analyzed airborne fungal exposure in mold-sensitive children with asthma living in urban areas in the United States. Fungal concentrations were measured in and around 414 homes across seven urban communities; common fungi, such as *Cladosporium, Penicillium, Aspergillus*, and *Alternaria,* were found. The study showed a strong correlation between indoor and outdoor fungal levels, with 50.5% of outdoor and 30.9% of indoor samples containing *Alternaria* spores. Outdoor spores substantially affected indoor air quality, as indoor fungal concentrations were moderately correlated with outdoor levels (r = 0.32, *p* < 0.0001). Homes with moisture issues had 1.9-times higher odds of elevated *Alternaria* levels, whereas cockroach-infested homes had 3.3-times higher odds of increased *Aspergillus* spores. Pet ownership, especially cats, was associated with a 2.3-fold increase in total fungal concentration. Conversely, homes with forced-air heating had 60% lower odds of high fungal levels, and winter sampling reduced *Aspergillus* and *Penicillium* concentrations by 50% [34]. In a study by Soffer et al., *A. alternata* was found in 85% of children’s homes in New York City, with higher levels associated with carpeting, wet mopping, and lower-income neighborhoods [35]. Baxi et al. investigated mold exposure in inner-city school classrooms attended by children with asthma. Airborne mold spores were measured in 180 classrooms across 12 schools, indicating the presence of mold in all classrooms, but with substantial variability in concentration and diversity between rooms within the same school. The most commonly detected mold types included *Cladosporium* and *Aspergillus*. *Alternaria* was found in 29% of the classrooms, with higher concentrations observed in rooms with visible mildew. Seasonal variations were also noted, with higher mold concentrations in the fall, highlighting the potential influence of environmental factors on mold growth in classrooms [36]. *Alternaria alternata* is the most common species in the *Alternaria* genus. A total of 16 allergens of *A. alternata* were identified (Alt 1, Alt 3, Alt 4, Alt 5, Alt 6, Alt 7, Alt 8, Alt 10, Alt 12, Alt 13, Alt 14 and Alt 15, Alt a 2, Alt a 9, Alt a NTF2 and Alt a TCTP). However, the World Health Organization (WHO)/International Union of Immunological Societies (IUIS) Allergen Nomenclature Subcommittee includes the first 14 molecules [7,37,38].

*Alternaria* allergens have various functions and exhibit high cross-reactivity with multiple allergens, including those from both fungal and non-fungal sources. Alt a 1, a major fungal protein of unknown function, shows structural similarities to allergens from *A. brassicola* (Alt b 1), *Ulocladium chartarum* (Ulo c 1), *Embellisia allii* (Emb a 1), *Nimbya celosiae* (Nim c 1), *Sinomyces fusoideus* (Sin fu 1), and *Stemphylium botryosum* (Ste b 1). Minor allergens, such as heat shock proteins (Alt a 3), ribosomal proteins (Alt a 5, 12), enolases (Alt a 6), glutathione transferases (Alt a 13), vacuolar serine protease (Alt a 15), and manganese superoxide dismutase (Alt a 14) exhibit cross-reactivity with homologous proteins from *Penicillium*, *Aspergillus*, *Cladosporium*, *Curvularia*, *Rhodotorula*, and *Malassezia* [7,27,39]. Moreover, Alt a 6, previously known as Alt a 5, shows cross-sensitivity with Hev b 9, the enolase of latex [40] (Table 2). The strong cross-reactivity of *Alternaria* allergens with multiple fungal species is responsible for polysensitization, leading to broader allergic responses and diagnostic challenges, and limiting the efficacy of allergen-specific immunotherapy (AIT).

### 2.2. Alternaria as an Inducer of Allergic Inflammation

Upon inhalation, *A. alternata* spores penetrate the upper and lower airways, where they come into contact with structural and immune cells, including airway epithelial cells, dendritic cells, and alveolar macrophages. These fungal components are recognized by pattern recognition receptors (PRRs), such as toll-like receptors (TLRs), TLR2, and TLR4, protease-activated receptor 2 (PAR-2), and the organic cation transporter, SLC22A17. This recognition leads to the activation of downstream signaling pathways and rapid release of epithelial-derived alarmins, including interleukin (IL)-33, IL-25, and thymic stromal lymphopoietin (TSLP) [41,42,43]. These alarmins play a pivotal role in skewing the immune response toward type 2 immunity. They activate group 2 innate lymphoid cells (ILC2s), which secrete IL-5 and IL-13; these are cytokines that respectively promote eosinophilic inflammation and enhance mucus hypersecretion and airway hyperresponsiveness (AHR), which are hallmarks of allergic asthma [44]. TSLP also influences dendritic cells, conditioning them to drive naïve CD4+ T cells toward a Th2 phenotype, thus linking the innate and adaptive immune responses.

Although allergic asthma has traditionally been characterized by Th2-type responses, *A. alternata* exposure may also elicit Th1- and Th17-mediated inflammation, depending on the host immune status and cytokine microenvironment. This is particularly relevant because antifungal immunity is typically dominated by Th1/Th17 responses, whereas allergic responses reflect a dysregulated Th2 axis. IFN-γ and TNF-α, products of Th1 cells, as well as IL-17 from Th17 cells, may contribute to airway inflammation and remodeling, complicating the immune landscape in fungal-induced asthma [44,45,46]. These immune responses are part of a broader spectrum of fungal-related respiratory conditions. Fungal lung diseases are heterogeneous and can be classified as infectious, toxic, or allergic diseases. Within the allergic domain, two major subtypes have been described [47]. The first involves sensitization to environmental fungi, such as *Alternaria* and *Cladosporium*, which act as seasonal aeroallergens, similar to grass pollen. Exposure correlates with ambient spore concentrations and may provoke acute asthma exacerbations, especially during the peak fungal seasons. The second subtype refers to sensitization to thermotolerant, filamentous fungi, such as *Aspergillus* and *Penicillium*, which are not only aeroallergens, but are also capable of colonizing the lower airways. This colonization creates persistent allergenic and inflammatory stimuli that may cause progressive lung damage.

*Alternaria* has been implicated in co-sensitization phenomena, acting as an adjuvant for other allergens such as grass pollen and food allergens, such as kiwifruits, peaches, and bananas. In particular, *Alternaria* spp. can act as allergic inducers (e.g., kiwifruit thaumatin-like proteins), further complicating allergy management. When *Alternaria* spores are placed on kiwifruit, they increase the expression of Alt a 1, which binds to kiwi PR5 (Act d 2), a plant defense protein with antifungal properties, and co-localizes within the fruit tissue [48]. This interaction inhibits the enzymatic activity of Act d 2, with several consequences. First, the inhibition of PR5 proteins weakens the plant’s natural antifungal defenses, making the fruit more susceptible to infections. Additionally, the interaction increases the risk of co-sensitization, making individuals allergic to *Alternaria* more likely to develop fruit allergies.

In conclusion, the presence of Alt a 1 in the fruit pulp, even in the absence of visible fungal growth, enhances fruit allergenicity and increases the likelihood of allergic reactions in sensitized individuals [42]. This phenomenon complicates allergy diagnosis and management, as patients may experience unexpected allergic responses to eating fruits that they have not previously reacted to.

## 3. Clinical Manifestations and Natural History of *Alternaria* Sensitization

Asthma and rhinitis are the most common symptoms of *Alternaria* allergy. Because the nasal mucosa represents a primary immunological and anatomical interface with inhaled environmental particulates and allergens, it is a frequent site of fungal deposition. Consequently, fungal species are often identified in nasal secretions [43]. AR affects approximately 40% of the population and is characterized by sneezing, nasal congestion, rhinorrhea, and nasal itching. Ocular symptoms are also common. Despite its benign nature, AR can impair sleep and cognitive function, substantially reducing quality of life [49].

Asthma is a chronic inflammatory airway disease affecting more than 300 million individuals worldwide. It imposes a substantial burden on healthcare systems, both economically and in terms of patient morbidity. The clinical presentation is heterogeneous and characterized by variable airflow limitation, airway inflammation, and symptoms such as dyspnea, wheezing, and chronic cough. The underlying pathophysiological mechanisms include bronchial hyperresponsiveness, smooth muscle contraction, mucus overproduction, and structural changes, such as airway remodeling, fibrosis, and epithelial thickening. These alterations can occur independently or in combination, contributing to the complexity of disease expression. As a result, distinct endotypes have been defined, including allergic asthma, which is closely associated with sensitization to environmental allergens, such as *Alternaria* [43,50,51].

The clinical manifestations of *Alternaria* allergy are best recognized in monosensitized individuals. In a pediatric study, 18 children monosensitized to *Alternaria* in a group of 1057 (1.7%) children seen in an allergy center were identified. Clinical manifestations were asthma in ten (55.5%), recurrent cough in six (33.3%), persistent rhinitis in four (22.2%), dermatitis in four (22.2%), and seasonal oculorhinitis in three (16%) children [52].

These findings were later confirmed by Cantani et al. in a prospective epidemiological study involving 6840 Italian children with asthma or allergic rhinitis. Of these, 213 (3.3%) were sensitized to *A. alternata*, whereas only 89 (1.3%) were monosensitized. Clinically, eighty-three children presented with asthma or allergic rhinitis, and six had atopic dermatitis [53]. Further supporting evidence comes from a population-based French study conducted as part of the ISAAC Phase II (Six Cities) study, which examined the relationship between *Alternaria* sensitization and allergic rhinitis. Of 6726 children (mean age: 10 y), *Alternaria* sensitization was found in 2.8%, with 0.8% being monosensitized. Sensitized children had a substantially higher prevalence of rhinoconjunctivitis. Importantly, even after excluding children with asthma, monosensitization to *Alternaria* remained strongly associated with both past year rhinoconjunctivitis and allergic rhinitis not caused by pollen, suggesting a direct role of *Alternaria* in upper airway disease independent of asthma [54].

Mold sensitization plays a key role in asthma exacerbation, increased emergency room visits, and respiratory distress [55,56,57]. Soffer et al. [35] found that sensitization to *A. alternata* was linked to a 3.7-fold increased risk of frequent wheezing and other symptoms of asthma. Moreover, *A. alternata* levels in house dust were associated with higher exhaled nitric oxide (FeNO) levels, particularly in areas with high environmental carbon levels, suggesting that combustion byproducts may worsen fungal-induced airway inflammation [35]. Baxi et al. [58] explored the relationship between fungal spore exposure in classrooms and asthma morbidity in inner-city schools as part of the School Inner-City Asthma Study (SICAS). The study involved 280 children (mean age, 7.9 y) with physician-diagnosed asthma, with 9.2% of the participants sensitized to *Alternaria*. *Alternaria* spores were detected in 22% of the classrooms. Children exposed to higher levels of *Alternaria* in the classroom experienced 3.2-times more asthma clinical manifestation days per 2-w period compared to those in low-exposure environments [58].

Geographic location also substantially affects the prevalence of *Alternaria* sensitization. A pediatric study comparing two climatically distinct Chinese cities, Lanzhou (dry and cold) and Wuhan (hot and humid), found higher asthma, AR, and mold exposure rates in Wuhan, particularly in suburban areas and among younger children. Household mold exposure was strongly associated with an increased risk of asthma (OR = 2.399), AR (OR = 1.969), and substance allergies (OR = 1.729) in Wuhan, highlighting the role of regional climate in mold-related health outcomes [59].

Moreover, early-life exposure to *Alternaria* spores has been shown to increase the risk of allergic sensitization and asthma, especially in genetically predisposed children [60]. The results from a high-risk cohort from infancy to 7-y evaluating mold exposure, including *Alternaria*, at both 1-y and 7-y of age, demonstrated that children exposed to high mold levels (high-ERMI homes) at 1 y were more than twice as likely to develop asthma by 7 y compared to children living in low-ERMI homes. Additional substantial risk factors identified included parental asthma and allergic sensitization to house dust mites [60,61]. Similar results were observed in The Isle of Wight birth cohort in the UK, where *Alternaria* sensitization at 4-y of age was associated with long-term risk of asthma, rhinitis, and lung function deficits from 10 to 26 y [62].

Moreover, exposure to airborne *Alternaria* spores was identified as a potential trigger of severe and potentially life-threatening asthma exacerbation, both in outdoor settings and during controlled allergen provocation tests [63]. Of 11 young patients with asthma who experienced sudden respiratory arrest, 91% showed positive skin tests for *Alternaria* versus only 31% of the matched asthma controls. Serum IgE levels to *Alternaria* were also elevated in affected individuals. All respiratory arrest episodes occurred during the peak mold season. After adjusting for age, *Alternaria* sensitization was associated with a nearly 200-fold increase in the risk of respiratory arrest [64].

## 4. Diagnosis of *Alternaria* Allergy

Diagnosing mold allergies can be challenging due to the variability in mold allergens and the decreasing availability of commercial test solutions. *Alternaria* extract exhibits the highest degree of standardization among mold allergens, meeting international standardization guidelines (Food and Drug Administration, European Medicines Agency, and WHO/IUIS) and ensuring reproducibility in allergy diagnostics and immunotherapy [65]. Some factors must be addressed in the diagnosis of *Alternaria* allergy.

**Figure 1:**
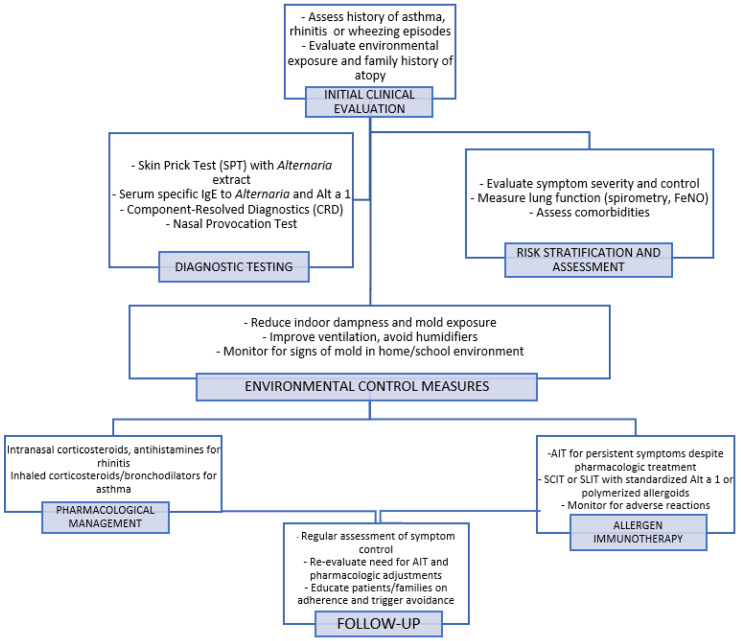
Diagnostic and management algorithm for *Alternaria alternata* allergy. The flowchart outlines steps from initial clinical evaluation and diagnostic testing to environmental control, pharmacological management, and allergen immunotherapy. It emphasizes a personalized approach including symptom monitoring and follow-up.

(1) Establishing a clear cause–effect relationship in *Alternaria* allergy is particularly challenging. Unlike other allergens, such as house dust mites or pollen, whose exposure patterns are more easily monitored and temporally linked to symptom onset, *Alternaria* spores exhibit high variability in both time and space. Their concentration in the air can fluctuate rapidly depending on weather conditions, humidity, and local vegetation, making exposure quantification inconsistent. *Alternaria* spores often coexist with other airborne allergens, such as grass or olive pollen, which are prevalent during the same seasons. This co-exposure complicates clinical interpretation, as patients frequently present with polysensitization, and symptoms may not be attributable to a single allergen. Consequently, even in sensitized individuals, it is difficult to definitively associate clinical manifestations with *Alternaria* exposure alone [7]. In addition, traditional environmental monitoring relies on total spore counts, which do not necessarily reflect the actual allergenic load. Symptoms correlate more closely with the levels of specific allergens such as Alt a 1 than with overall spore counts [66]. This discrepancy further impairs the ability to directly link exposure to symptom exacerbation.

(2) In the diagnosis of mold allergy, the skin-prick test (SPT) is more sensitive than specific IgE (sIgE) in detecting mold sensitization, with the exception of *Alternaria* [67]. Kespohl et al. examined 168 patients with mold exposure or mold-induced respiratory symptoms, assessing SPT solutions biochemically, and applying them in duplicate on the patients’ arms. The sIgE levels were measured using ImmunoCAP. The SPT identified mold sensitization more frequently (90/168) than sIgE testing (56/168). *Alternaria* represented an exception, showing the highest sensitization prevalence, with 46 patients (27%) testing positive using sIgE and up to 60 (36%) testing positive using SPT, depending on the manufacturer. Concordance among different SPT solutions varied, but for Alt a, the agreement was the highest, with 69% (40 of 58) of sensitized individuals reacting to all tested SPT solutions. The high concordance was between sIgE and SPT for *Alternaria*, with an area under the curve (AUC) of 90.1–91.6% in the receiver operating characteristic analysis, indicating that sIgE for *Alternaria* was as reliable as SPT, unlike other mold allergens, where SPT demonstrated superior sensitivity [68]. Similarly, the study by Fernández et al. demonstrated that both SPT and serum-specific IgE (sIgE) were highly effective in predicting bronchial reactions to *Alternaria* in asthmatic patients; of 74 sensitized individuals, 61% had a positive bronchial challenge. SPT showed excellent predictive accuracy (AUC, 0.957), with a 5.5 mm wheal indicating a 90% likelihood of a positive response and sIgE levels ≥16 kUA/L predicting a positive reaction with 99% accuracy [69].

(3) Component-resolved diagnosis (CRD) can differentiate true allergen sensitization from cross-reactions, improving diagnosis and guiding immunotherapy choices. In the diagnostic process, Alt a 1, the major A. *alternata* allergen, is strongly correlated with clinical manifestations (over 80–90% of patients show sensitivity to this allergen), SPT, and sIgE levels [70,71]. Therefore, SPT and sIgE testing are recommended for patients with suspected allergies, whereas CRD should be reserved for those considered for AIT [72].

(4) The provocation test plays a key role in identifying clinically suspected cases that yield negative skin and laboratory test results. Krouse et al. evaluated the efficacy of epicutaneous and intradermal testing in predicting the nasal response to *Alternaria* antigen in the nasal provocation test (NPT). The sensitivity and specificity of epicutaneous testing were low (42% and 44%, respectively), with intradermal testing only marginally increasing the sensitivity to 58% [73]. Similarly, Kupczyk et al. found that NPT results were not correlated with sIgE levels, SPT results, or seasonal symptoms in children and adolescents allergic to *Alternaria* [74]. Fuiano et al. measured the nasal IgE levels in children with rhinitis during high *Alternaria* spore exposure periods. Of the 56 children studied, 37.5% tested positive on SPT and 80.3% tested positive on nasal IgE testing (NT). A total of 64.3% of the children with negative SPT results had positive NT results, indicating local IgE production. Furthermore, 69.6% of NT-positive cases showed a positive NPT response, compared to only 26.8% of SPT-positive cases (*p* < 0.0001) [75]. This discrepancy suggests that the standard diagnostic tests may not fully capture the complex mechanisms underlying *Alternaria* sensitization, potentially leading to inaccurate or overlooked diagnoses. In particular, *Alternaria* has been identified as a trigger for local AR (LAR), a distinct AR subtype characterized by localized allergic inflammation in the nasal mucosa without systemic sensitization [76]. Unlike classical AR, which is confirmed by detectable allergen-specific IgE in blood tests or positive SPT results, patients with LAR exhibit allergen-specific IgE production exclusively within the nasal mucosa. Utilizing NPT and local sIgE testing can aid in accurately diagnosing cases that may have been misclassified as non-AR (NAR) but are, in fact, indicative of LAR [77,78,79]. LAR is managed similarly to AR with treatments that include allergen avoidance, medication, and AIT [76,80,81]. The diagnostic and management algorithms for *Alternaria alternata* allergy are shown in Figure 1.

Diagnostic criteria have been suggested for specific clinical conditions, including clinical manifestations and allergological, laboratory, and instrumental tests to direct the diagnosis toward a fungal form. For allergic fungal rhinosinusitis (AFRS), Bent and Kuhn diagnostic criteria have been proposed. These include the presence of chronic rhinosinusitis symptoms lasting for more than 12 w, such as nasal congestion, facial pain, nasal discharge, and anosmia. Bilateral nasal polyps must be observed during nasal endoscopy, and eosinophilic inflammation in the sinus tissue must be observed via histopathological examination. Fungal elements, such as hyphae or spores, must be identified in the sinus tissue through histology, direct culture, or polymerase chain reaction (PCR) testing. Positive allergy tests and CT scan findings of mucosal thickening, sinus opacification, and fungal debris are essential. Finally, other causes of chronic rhinosinusitis, especially non-fungal infections, must be ruled out. A patient must meet all five criteria to be definitively diagnosed with AFRS [82].

*Alternaria* is a common fungal genus implicated in non-*Aspergillus* allergic bronchopulmonary mycosis (ABPM). A definitive diagnosis of ABPM requires meeting at least six of the established criteria. The patient must have a history of asthma or asthma-like clinical manifestations, with peripheral blood eosinophilia exceeding 500 cells/µL and total IgE levels > 417 IU/mL. Sensitization to filamentous fungi (e.g., *Alternaria*, *Curvularia*, *Bipolaris*, *Cladosporium*) must be confirmed through positive-specific IgE or skin testing, whereas specific IgG or precipitating antibodies further support the diagnosis. Imaging findings should indicate central bronchiectasis and/or high-attenuation mucus on chest CT scans. Additionally, laboratory tests may identify a positive fungal culture from the bronchial mucus or bronchoalveolar lavage. Clinically, the disease often manifests as recurrent expectoration of mucus plugs and migrating pulmonary infiltrates. A definitive diagnosis is confirmed if at least six criteria are met, whereas five criteria suggest a suspected diagnosis [83].

Finally, the diagnosis of severe asthma with fungal sensitization (SAFS) requires persistent, difficult-to-control asthma with frequent exacerbations despite optimal treatment. These patients typically have a history of poor asthma control and reduced lung function. Fungal sensitization is confirmed by positive allergy tests, such as SPT or serum-specific IgE measurements. Additionally, evidence of fungal exposure, including a history of allergic fungal rhinosinusitis or environmental mold exposure, supports this diagnosis. An elevated eosinophil count in peripheral blood or sputum indicates an underlying eosinophilic inflammatory response. Before confirming SAFS, other potential triggers of asthma exacerbation, such as viral infections or non-fungal allergens, must be ruled out. Treatment strategies typically involve high-dose inhaled corticosteroids, biological therapies, and antifungal medications, depending on the severity and extent of fungal involvement [84] (Table 3).

## 5. Treatment of *Alternaria* Allergy

Interventions for *Alternaria* spp. allergy require a multifaceted and individualized approach that includes environmental control strategies, pharmacological treatments, and AIT in selected cases. The primary goal is to reduce allergen burden and mitigate the associated inflammatory responses that manifest as AR, conjunctivitis, or asthma. Minimizing the exposure to fungal allergens is a cornerstone of management. This involves the identification and remediation of indoor sources of moisture and mold growth, ensuring adequate ventilation, repairing water damage, and maintaining indoor humidity below 50%. The use of dehumidifiers and high-efficiency particulate air (HEPA) filters may help reduce airborne fungal spore concentrations, especially in sensitized individuals [85,86,87].

The pharmacological management of fungal-induced AR and conjunctivitis does not differ substantially from the treatment protocols used for other aeroallergens, such as pollens, house dust mites, or animal dander. According to the Allergic Rhinitis and Its Impact on Asthma (ARIA) guidelines, intranasal corticosteroids are considered the first-line treatment because of their strong anti-inflammatory efficacy. In cases where symptom control remains inadequate, combination therapy may be warranted, incorporating second-generation oral antihistamines, intranasal antihistamines, typical antihistamines, or mast cell stabilizers when ocular involvement is present, topical antihistamines, or mast cell stabilizers [41,49]. In individuals sensitized to fungal allergens, including *Alternaria*, asthma management aligns with the stepwise pharmacological approach recommended by the Global Initiative for Asthma (GINA). Treatment is tailored to the level of symptom control and severity, beginning with the use of inhaled corticosteroids as foundational therapy. If control remains insufficient, escalation includes the addition of long-acting β2-agonists or leukotriene receptor antagonists, following GINA recommendations [51].

### Allergen Immunotherapy to Alternaria

AIT is a disease-modifying treatment that involves the administration of increasing doses of specific allergens to induce immune tolerance [88,89]. Although specific data on its preventive role in *Alternaria* allergy are limited, the rising prevalence of sensitization in children and the typical progression from early exposure to rhinitis and eventually to asthma suggest that AIT could have a potential role in modifying the natural course of the disease and reducing the risk of long-term complications [90]. This fungus has been standardized over the past 35 years, and recombinant extracts are now utilized in clinical practice. Generally, immunotherapy with *A. alternata* extract has shown positive results, substantially reducing clinical manifestations and medication use compared to other models. Treatment tolerance is usually good, with minimal adverse reactions [90,91,92,93,94,95]. AIT for *A. alternata* involves subcutaneous immunotherapy (SCIT) and sublingual immunotherapy (SLIT).

Horst et al. conducted a double-blind, placebo-controlled (DBPC) clinical trial investigating the efficacy and safety of SCIT with a standardized *A. alternata* extract in patients with allergic rhinitis and/or asthma, including pediatric patients as young as 5 y (age range: 5–56 y). A total of 24 patients were enrolled, with 13 receiving active SCIT and 11 receiving the placebo. Treatment was administered using a rush protocol, followed by maintenance injections. Throughout the study, the patients in the SCIT group demonstrated substantial clinical improvements, including reduced symptom scores, decreased medication use, reduced skin reactivity to *Alternaria*, and increased tolerance to nasal allergen challenges. Immunologic responses include an increase in *Alternaria*-specific IgG levels. The treatment was generally well tolerated, although two mild asthma exacerbations occurred during the rush induction phase. These resolved without requiring discontinuation of therapy [96].

An RCT by Kuna et al. evaluated the efficacy and safety of SCIT with a standardized *A. alternata* extract in children and adolescents allergic to this mold. Fifty patients (ages 5–18 y) with *A. alternata*-induced AR and/or asthma were randomly assigned to receive either active treatment or placebo. After 3 y, the combined symptom medication scores decreased substantially by 38.7% in year two and 63.5% in year three (*p* < 0.001). This improvement was linked to a better quality of life and decreased sensitivity to the nasal allergen challenge. Mild side effects such as injection site edema were noted in seven patients [97]. The study by Kiliç et al. enrolled 16 children with bronchial asthma and *Alternaria* monosensitization, divided into an immunotherapy group (Group I, nine patients) and a control group (Group II, seven patients) [98]. One year after SCIT, Group I showed a substantial reduction in bronchial responsiveness to methacholine and *Alternaria* (*p* = 0.03, *p* = 0.006, respectively) compared to controls. Specific IgE levels were decreased in the immunotherapy group (*p* = 0.001). Following allergen provocation, sputum eosinophil counts were lower in the SIT group (*p* = 0.011), whereas sputum eosinophil cationic protein (ECP) levels did not change in the SIT group, but increased substantially in the control group.

An RCT by Tabar et al. evaluated the efficacy and safety of SCIT with two doses of Alt a 1, the major allergen of *A. alternata*, in adolescent and adult patients with rhinoconjunctivitis caused by *A. alternata* sensitization, with or without controlled asthma [99]. The active groups received either 0.2 μg or 0.37 μg of Alt a 1, administered subcutaneously, and the primary endpoint was the combined symptom and medication score. The treatment schedule included an induction phase with four weekly doses every 7 d, followed by one dose after 15 d, and a maintenance phase with one dose administered approximately every 30 d. Results showed substantial reductions in symptoms and medication use in the 0.37-μg group compared to the placebo after 12 months. Both the active groups demonstrated improved cutaneous reactivity, reduced IgE levels, and increased IgG4 levels. The safety profile was comparable across all groups with no serious adverse reactions [99].

To date, RCTs on the use of SLIT for *Alternaria* have been conducted predominantly in adults and have demonstrated substantial results for both AR and asthma [100,101].

Liu et al. reported the largest pediatric cohort treated with SCIT for *A. alternata*-induced respiratory allergies in China [102]. Of the 70 patients (aged 5–27 y), nearly half were children aged 5–11 y, and 31 received monotherapy with *A. alternata* extract. After at least 1 y of SCIT, the authors observed substantial clinical improvements, including reductions in total symptom scores (RTSS), medication use (RMS, CSMS), and enhanced asthma control (ACT). Additionally, spirometric parameters, such as FEV1%, MEF25, and PEF, improved substantially. Younger children (5–11 y) showed a better response to the combined symptom and medication scores than older patients. The therapy was well tolerated and no serious adverse events were reported [102] (Table 4).

*Alternaria* AIT faces challenges, such as inconsistent allergen dosages, adverse reactions, and prolonged treatment durations. The complexity of *A. alternata* allergenic proteins and the potential for adverse effects of native fungal extracts complicate treatment. Alternative strategies, such as hypoallergens or purified allergens (Alt a 1), show promise, but require further research to confirm their long-term safety and effectiveness. Research is increasingly focusing on *A. alternata* allergoids, which appear to offer safer and more stable alternatives to traditional immunotherapy, providing enhanced effectiveness and reducing the need for injections in polysensitized patients [103]. Brindisi et al. investigated the efficacy of SCIT using a polymerized Alt a 1 allergoid in children monosensitized to *A. alternata* with persistent AR and intermittent asthma [104]. A total of 42 children were enrolled, of whom 17 received active treatment and 25 continued symptomatic therapy. Various tests were conducted at the start (T0) and after 24 months (T1), including total IgE (tIgE), specific IgE (sIgE) for Alt a 1, nasal nitric oxide (nFeNO), nasal cytology, anterior active rhinomanometry, and spirometry. The results indicated that patients treated with allergoids showed substantial improvements, including a decrease in nFeNO, tIgE, and sIgE for Alt a 1, nasal eosinophil counts, and an increase in nasal airflow and FEV1. Specifically, there was a substantial reduction in allergic symptoms, with improvements observed both objectively and through laboratory measurements [104].

Future research should aim to enhance the efficacy of AIT by improving allergen standardization, optimizing treatment protocols, and identifying biomarkers that can predict treatment success. Given its potential to provide long-term relief and disease modification, AIT remains a crucial therapeutic option for *Alternaria*-induced allergic diseases in both.

**Table 4:** Clinical studies evaluating allergen immunotherapy (AIT) for *Alternaria alternata* in pediatric patients, either exclusively or as part of mixed-age cohorts that included children.

Study	Design & Participants	AIT Type	Main Findings	Safety Profile	Comment
Horst 1990 [96]	DBPC; 24 patients (5–56 yrs); 13 SCIT, 11 placebo	SCIT (standardized via RI)	↓ Symptoms and medication, skin reactivity; ↑ nasal challenge dose, specific IgG (*p* < 0.001)	2 mild asthma reactions during rush protocol.	Well-controlled DBPC study demonstrating both clinical and immunologic efficacy; limited by small sample and use of rush protocol, which may not reflect standard clinical practice.
Kuna 2011 [97]	RCT; 50 children/adolescents (5–18 yrs) with *A. alternata*-induced AR and/or asthma; placebo-controlled	SCIT (standardized extract)	↓ Symptoms: −38.7% (year 2), −63.5% (year 3); ↑ QoL; ↓ nasal allergen sensitivity	Mild (injection site oedema in seven pts).	Robust RCT with long-term follow-up demonstrating sustained clinical efficacy and improved quality of life; limited by small sample size and absence of mechanistic biomarker analysis.
Kiliç 2011 [98]	16 children with asthma and *Alternaria* mono-sensitization; Group I (9) SCIT, Group II (7) control	SCIT	↓ Bronchial reactivity; ↓ sIgE (*p* = 0.001); ↓ sputum eosinophils (*p* = 0.011); ↑ ECP in controls	Mild local reactions (injection site erythema and swelling in three patients); no systemic or severe adverse events reported.	Controlled prospective design; extensive clinical and immunologic endpoints (sIgE, ECP, sputum eosinophils, bronchial challenges); demonstrated early improvements in airway inflammation. Small sample size; short duration (1 year), not controlled-randomized study.
Tabar 2019 [99]	RCT; adolescents/adults with *Alternaria*-induced rhino-conjunctivitis ± asthma; 0.2 µg and 0.37 µg Alt a 1 vs. placebo	SCIT (Alt a 1)	0.37 µg group: ↓ symptoms/medication; ↓ IgE, ↑ IgG4; ↓ skin reactivity	The most common adverse events were local reactions at the injection site, which were mild and transient.	Well-designed RCT using a recombinant allergen with dose-response analysis; showed immunological and clinical benefits. Limited by short duration (12 months) and lack of long-term efficacy data.
Liu 2024 [102]	31 subjects (mean age 12.03 ± 4.32; 42% 5–11 years) with perennial AR and/or asthma	SLIT (Alt a 1)	↓ Symptoms and meds; ↓ nasal eosinophils; ↑ nasal flow; effective even in polysensitized	Well tolerated; no serious effects.	Real-world pediatric data supporting SLIT efficacy in AR/asthma, even in polysensitized patients; limited by retrospective design, short follow-up (1 year), and lack of control group.
Brindisi 2023 [104]	42 children with AR ± asthma; 17 received polymerized Alt a 1 allergoid, 25 controls	SCIT (polymerized Alt a 1 allergoid)	↓ nFeNO, tIgE, sIgE; ↓ nasal eosinophils; ↑ airflow, FEV1; improved clinical/lab outcomes	No serious adverse effects; treatment was well tolerated without systemic reactions.	Prospective pediatric study showing clinical, functional, and immunologic improvements with polymerized Alt a 1 SCIT; limited by small sample size and lack of blinding.

Abbreviations: AIT = Allergen Immunotherapy; AR = Allergic Rhinitis; SCIT = Subcutaneous Immunotherapy; SLIT = Sublingual Immunotherapy; RCT = Randomized Controlled Trial; DBPC = Double-Blind Placebo-Controlled; QoL = Quality of Life; sIgE = Specific Immunoglobulin E; tIgE = Total Immunoglobulin E; IgG4 = Immunoglobulin G4; ECP = Eosinophil Cationic Protein; nFeNO = Nasal Fractional Exhaled Nitric Oxide; FEV1 = Forced Expiratory Volume in 1 Second; SPT = Skin Prick Test; RI = Rush Immunotherapy.

## 6. Conclusions

Molds, including *A. alternata*, represent a key component of human exposure and internal mycobiota, with sensitization prevalence influenced by geography and environmental exposure. *Alternaria alternata* is one of the most common mold allergens affecting both pediatric and adult populations. Although predominantly found in outdoor environments, it can colonize damp indoor settings, such as poorly ventilated buildings. Airborne spore concentrations, which are typically highest from May to November, are increasingly affected by climate change. Sixteen allergens were identified, of which Alt a 1 was the most clinically relevant. *Alternaria alternata* may also act as an adjuvant to enhance co-sensitization to other allergens. Asthma and allergic rhinitis are the most frequent clinical manifestations; however, diagnosis remains challenging due to allergen variability and limitations in diagnostic tools. Allergen immunotherapy via both sublingual and subcutaneous routes offers a targeted strategy for reducing symptoms and improving patient outcomes in both adults and children (Table 5).

## Figures and Tables

**Table 1:** Reported prevalence rates of sensitization to *Alternaria alternata* in various countries and regions. Data reflect studies conducted in general populations or among patients with respiratory allergies, highlighting substantial geographic variability.

Country/Region	Prevalence (%)
USA	16.6% [11]
Europe	~5% (confirmed by skin tests) [12,13,14]
Greece	23.8% among allergic patients [18]
Germany	10.6% among allergic patients [18]
Finland	10.3% among allergic patients [18]
France	10.3% among allergic patients [18]
Denmark	8.2% among allergic patients [18]
Austria	6.5% among allergic patients [18]
Hungary	6.5% among allergic patients [18]
Belgium	6.2% among allergic patients [18]
Netherlands	5.5% among allergic patients [18]
Portugal	5.5% among allergic patients [18]
Switzerland	5.5% among allergic patients [18]
Italy	3.5% among allergic patients [18]; 10.4% among allergic patients [19]
Poland	3.5% among allergic patients [18]
United Kingdom	0.8% among allergic patients [18]
Spain	20.2% among allergic patients [20]
Peru (Lima)	7% [21]
Gabon (Africa)	15% [22]
Iran	5.3% among allergic patients [23]
China (asthmatic children)	14.9% (with asthma) 44.9% (with AR) [24]
Samara (Russia)	42.2% (children with AR and asthma) [25]

AR: allergic rhinitis.

**Table 2:** Summary of major and minor *Alternaria alternata* allergens, their functions, and molecular weights.

Allergen	Protein Type/Biological Function	MW (kDa)	Clinical Relevance
Alt a 1	Dimeric β-barrel protein, fungus-specific	15.3/16.4	Major allergen
Alt a 3	Heat shock protein 70	85	Minor allergen
Alt a 4	Disulfide isomerase	57	Minor allergen
Alt a 5	Ribosomal protein P2	11	Minor allergen
Alt a 6	Enolase	45	Minor allergen
Alt a 7	Flavodoxin-like YCP1 protein	22	Minor allergen
Alt a 8	Mannitol dehydrogenase	29	Minor allergen
Alt a 10	Aldehyde dehydrogenase	53	Minor allergen
Alt a 12	Acidic ribosomal protein P1	11	Minor allergen
Alt a 13	Glutathione-transferase	26	Minor allergen
Alt a 14	Manganese superoxide dismutase (Mn-SOD)	24	Minor allergen
Alt a 15	Vacuolar serine protease	58	Minor allergen
Alt a NTF2	Nuclear Transport Factor	14	Minor allergen
Alt a TCTP	Cytokine-like action	ND	Minor allergen

ND: not defined.

**Table 3:** Diagnostic criteria for fungal-associated allergic airway diseases.

Condition	Diagnostic Criteria	Key Notes
AFRS (allergic fungal rhinosinusitis)	1. Symptoms of chronic rhinosinusitis > 12 w (e.g., nasal congestion, facial pain, discharge, anosmia) 2. Bilateral nasal polyps on endoscopy 3. Eosinophilic inflammation on histopathology 4. Fungal elements in sinus tissue (via histology, culture, or PCR) 5. Positive allergy tests + CT findings (e.g., mucosal thickening, opacification, fungal debris)	All five criteria required for definitive diagnosis. Rule out non-fungal causes.
ABPM (allergic bronchopulmonary mycosis)	1. History of asthma or asthma-like symptoms 2. Eosinophils > 500 cells/μL 3. Total IgE > 417 IU/mL 4. Sensitization to filamentous fungi (e.g., *Alternaria*) confirmed by skin test or specific IgE 5. Positive specific IgG or precipitating antibodies 6. Central bronchiectasis and/or high-attenuation mucus on chest CT 7. Positive fungal culture (sputum or BAL) 8. Recurrent mucus plug expectoration 9. History of migrating pulmonary infiltrates 10. Exclusion of other causes	≥Six criteria = definitive diagnosis Five criteria = suspected diagnosis
SAFS (severe asthma with fungal sensitization)	1. Persistent, severe asthma with frequent exacerbations 2. Positive fungal sensitization (SPT or specific IgE) 3. History of poor asthma control and reduced lung function 4. Evidence of fungal exposure (e.g., AFRS or environmental mold) 5. Elevated eosinophil count in blood or sputum 6. Exclusion of non-fungal asthma triggers	Diagnosis is clinical and based on exclusion. Treatment includes high-dose ICS, biologics, antifungals.

ICS: Inhaled Corticosteroids, PCR: Polymerase Chain Reaction, CT: Computed Tomography, BAL: Bronchoalveolar Lavage, SPT: Skin Prick Test.

**Table 5:** Overview of key characteristics of *Alternaria alternata* allergy.

Topic	Key Information
Habitat	Mainly outdoors; also indoors in damp, poorly ventilated spaces
Seasonal Presence	May–November; influenced by climate change
Sensitization Prevalence	Varies with environment and geography
Common Allergen	*Alternaria alternata*, especially Alt a 1
Immune Response	Activates epithelial cells and macrophages upon inhalation
Co-sensitization	Acts as an adjuvant enhancing other allergen responses (grass pollen, fruits)
Main Symptoms	Asthma and allergic rhinitis
Diagnosis	Clinical history, SPT, sIgE, Component-Resolved Diagnostics (CRD), nasal provocation test (key in uncertain cases)
Prevention	Minimize exposure through environmental control: reduce indoor humidity (<50%), ventilate damp spaces, eliminate mold-contaminated materials; avoid outdoor activities during peak spore seasons (dry, windy days).
Treatment	Intranasal corticosteroids, antihistamines for rhinitisInhaled corticosteroids, bronchodilators for asthma AIT

## Abbreviations

The following abbreviations are used in this manuscript:

IgE	Immunoglobulin E
AR	Allergic Rhinitis
AIT	Allergen Immunotherapy
EMA	European Medicines Agency
FDA	Food and Drug Administration
WHO	World Health Organization
IUIS	International Union of Immunological Societies
TLR	Toll-Like Receptor
TSLP	Thymic Stromal Lymphopoietin
IL	Interleukin
IFN-γ	Interferon gamma
TNF-α	Tumor Necrosis Factor alpha
PR5	Pathogenesis-related protein 5
FeNO	Fractional exhaled Nitric Oxide
ERMI	Environmental Relative Moldiness Index
ISAAC	International Study of Asthma and Allergies in Childhood
SPT	Skin Prick Test
sIgE	Specific Immunoglobulin E
CRD	Component-Resolved Diagnosis
AUC	Area Under the Curve
NPT	Nasal Provocation Test
NT	Nasal Testing
LAR	Local Allergic Rhinitis
NAR	Non-Allergic Rhinitis
AFRS	Allergic Fungal Rhinosinusitis
ABPM	Allergic Bronchopulmonary Mycosis
SAFS	Severe Asthma with Fungal Sensitization
ICS	Inhaled Corticosteroids
BAL	Bronchoalveolar Lavage
SCIT	Subcutaneous Immunotherapy
SLIT	Sublingual Immunotherapy
ECP	Eosinophil Cationic Protein
FEV1	Forced Expiratory Volume in 1 s
nFeNO	Nasal Fractional Exhaled Nitric Oxide
